# Assessment of malnutrition in patients with liver cirrhosis using protein calorie malnutrition (PCM) score verses bio-electrical impedance analysis (BIA)

**DOI:** 10.1186/s13104-018-3640-y

**Published:** 2018-08-02

**Authors:** Om Parkash, Wasim Jafri, S. M. Munir, Romaina Iqbal

**Affiliations:** 1 Section of Gastroenterology, Department of Medicine, Aga Khan University Karachi, Stadium Road, Karachi, Pakistan; 2 Department of Medicine and Community Health Sciences, Aga Khan University Karachi, Karachi, Pakistan

**Keywords:** Cirrhosis, Malnutrition, Bioelectrical impedance analysis

## Abstract

**Objective:**

Malnutrition is a common problem in patients with liver cirrhosis and tools for nutritional assessment are under debate. We conducted this study to assess prevalence of malnutrition in cirrhotic patients using PCM score and BIA. Additionally we compared BIA to PCM score for detecting malnutrition in this patient population.

**Results:**

This was a cross sectional study conducted in two tertiary care hospitals of Karachi Pakistan on adults with liver cirrhosis. Malnutrition was assessed by PCM score using anthropometric measurements and biological specimens and (ii) Body cell mass was assessed using BIA. Malnutrition as estimated by the PCM score was present in 122 (73%) of patients in which most patients had mild malnutrition (n = 72 (45%)), followed by 34 (21%) with moderate malnutrition and 3 (1.9%) with severe malnutrition. Malnutrition according to BIA estimated through body cell mass could detect it in 98 (61%) of patients. There was optimal correlation of PCM score with body call mass (Pearson correlation coefficient = 0.3 (p value 0.001)). We conclude that majority of the patients with liver cirrhosis had malnutrition as determined by PCM score. BIA underscored the malnutrition in this patient population.

## Introduction

Cirrhosis is the late and irreversible stage of hepatic fibrosis, which is characterized by destruction of hepatic architecture and the development of nodules [1]. Almost 65–90% patients with advanced cirrhosis have malnutrition [2], which itself is an independent predictor of mortality in patients with end stage liver disease [3]. In Pakistan, the most common causes of cirrhosis are hepatitis C virus (HCV) and hepatitis B virus (HBV) [4, 5]. Studies focusing on malnutrition in cirrhosis due to viral etiology are limited. Therefore it is essential to assess malnutrition in this patient population of cirrhosis due to Hepatitis B and C, whose disease course is more complicated due to infective etiology, and different types of treatment.

BIA is a simple, noninvasive, inexpensive and quick method to estimate BCM [6], and it has also been used in patients with cirrhosis [7–9]. Decreased body cell mass is an indicator of malnutrition, cachexia, dehydration [10]. Anthropometric measures include Triceps skin fold thickness (TCF), Mid Arm Muscle Circumference (MAMC), Mid Arm Circumference [11] and height. There is still a lot of debate on which is the better tool for assessment of malnutrition, as reliable nutritional assessment of the cirrhotic patients is difficult due to ascites and edema [12]. Therefore we conducted this study to assess prevalence of malnutrition in cirrhotic patients using PCM and BIA (using BCM). Additionally we compared BIA to PCM score for detecting malnutrition in this patient population.

## Main text

### Methods

This was a cross sectional study conducted in the outpatient medicine clinic of Aga Khan University hospital Karachi (AKUH) and Jinnah postgraduate medical center (JPMC) Karachi. All patients aged ≥ 14 years, with either history of viral CLD (HBsAg or HCV) or non-viral CLD who had established diagnosis of liver cirrhosis of any etiology were recruited in the study after obtaining the informed consent. Consent from parent or guardian was taken for those who were minors. Liver cirrhosis was diagnosed based on ultra-sonographic evidence of chronic liver disease including shrunken liver, dilated portal vein, splenomegaly. We used nonrandom purposive technique for recruiting participants in this study [13, 14].

Study measurements included demographic information, measurement of anthropometric measures, history of decompensation (including upper gastrointestinal bleed, ascites, and portosystemic encephalopathy) biological specimens (urine and blood) and assessment of total body water and fat free mass using BIA.

#### Anthropometry

Height was measured with portable stadiometer to the nearest of 0.1 cm and mean of three readings were documented. Triceps skin fold thickness was measured with a Lange caliper [15]. Mid arm circumference was calculated from the right arm at mid-point equidistant from the acromion and olecranon, with the patient in the upright position and arm flexed at 90°. The arm muscle circumference was calculated by the following formula using MAMC = MAC − (TSF × 0.3142). Reference for MAMC was obtained from Indian study [16, 17]. Weight was measured on Tanita weighing scale to the nearest of 0.1 kg. Biological specimen including albumin, creatinine, lymphocyte and 24 h urinary creatinine was measured using ADVIA 1800 in the lab. Malnutrition was assessed by using the following formula for PCM score [18]

$${\text{PCM}} = \frac{{\% \;{\text{TCF}} + \% \;{\text{MAC}} + \% \;{\text{MAMC}} + \% \;{\text{lymphocyte}} + \% \;{\text{albumin}} + \% \;{\text{CHI}}}}{6}$$

where TCF is triceps skin fold, MAC is mid arm circumference, MAMC is arm muscle circumference. Percent TCF % MAC, % MAMC, % lymphocyte, % albumin and % CHI were calculated as percent of the normal values. These normal values from a healthy Indian population were; mean TCF = 12 cm, mean MAMC = 26 cm) were used for the above percent calculation [17]. Malnutrition was classified based on this score as, mild (99.9–80%), moderate (60–79.9%) and severe (< 60%) according to the recommendation by Blackburn et al. [19, 20]. PCM score for this study was considered as gold standard.

#### Bioelectrical impedance measurement

BIA was performed using a BIA 2000M (Data Input GmbH, Darmstadt, Germany) [21] applying alternating current of 800 micro amperes at 50 kHz in the clinic. BIA was measured in the supine position with arm and legs abducted from the body in the morning after an overnight fast. The two electrodes (one for sensor and other for source) were placed on the dorsum of both hand foot of the dominant side of the body. Resistance (R), reactance (Xc) and the phase angle (alpha) was measured at each frequency. All impedance measurements were taken with the patient supine, arms relaxed at the sides but not touching the body. Total body water (TBW) and fat free mass [22] was calculated by using formula by Kushner and Schoeller [23]. Body cell mass was calculated by the formula; BCM = F.F.M * 0.29 * LN(5.28). This was used for assessment in cirrhotic young adults [7]. Body cell mass should be at least 40% of the body weight for a person to be designated as not malnourished [24].

## Results

Approximately 200 patients were invited to participate, out of which 161 (response rate = 80%) patients with liver cirrhosis were enrolled. The reasons for not participating was inability to come back for an outpatient visit due to a distant residence outside the city. There were 76 (47.2%) males and mean age was 49.1 (11) years. Hepatitis B or C were the cause of cirrhosis in 138 (87.8%) patients while in 23 (14%) patients these markers were negative. There were 61 (37.9%) patients in child class A, 60 (37.3%) in child class B and 17 (10.6%) in child class C.

### Malnutrition

Overall and comparison of malnutrition by PCM score and BIA measurements in cirrhotic patients with and without Malnutrition is shown in Table 1. Malnutrition as estimated by the PCM score was present in 122 (73%) of patients in which most patients had Mild Malnutrition (n = 72 (45%)), followed by 34 (21%) moderate malnutrition and only 3 (1.9%) severe malnutrition (Table 2).

**Table 1:** Comparison of PCM measurements in cirrhotic patients overall, with and without malnutrition

Variables	Overall	Malnutrition by PCM score^a^		p value
	N = 161	Yes N = 122	No N = 39	
		Mean [27]	Mean [27]	
BMI (kg/m^2^)	22.01 (6.4)	21.1 (6.5)	25.0 (5.0)	0.002
Midarm circumference (cm)	26.4 (4.1)	25.4 (3.9)	29.1 (3.5)	< 0.001
Triceps skin fold (mm)	27.3 (10.4)	24.7 (9.9)	35.0 (7.7)	< 0.001
Muscle arm circumference (cm)	17.7 (3.24)	17.7 (3.2)	25.0 (0.9)	0.87
24 h Urinary creatinine (mg/dl)	0.80 (0.40)	0.7 (0.35)	1.1 (0.34)	< 0.001
Serum creatinine (mg/dl)	0.9 (0.6)	0.93 (0.53)	1.04 (0.99)	0.53
Serum albumin (g/dl)	3 (0.7)	2.9 (0.7)	3.2 (0.6)	0.004
Absolute lymphocyte count	20.1 (16.5)	17.3 (15.1)	32.3 (17.8)	0.01
Creatinine height index	0.06 (0.02)	0.05 (0.02)	0.08 (0.02)	0.08
PCM score^b^	81.14 (14.7)	84.0 (11.9)	112 (8.9)	< 0.001
Severity of malnutrition^c^				
Mild malnutrition (%)	–	72 (44.7)		
Moderate malnutrition (%)	–	34 (21.1)		
Severe malnutrition (%)	–	3 (1.9)		

^a^Malnutrition by PCM score is a score < 100% of PCM score (Blackburn et al.), missing data for 13 participants as they did not returned back with biological specimen

^b^ $${\text{PCM}}\;{\text{score}}\, = \,{\text{PCM}}\, = \, \frac{{\% \;{\text{TCF}}\; + \;\% \;{\text{MAC}}\; + \; \% \;{\text{MAMC}}\; + \; \% \;{\text{lymphocyte}}\; + \;\% \;{\text{albumin}}\; + \;\% \;{\text{CHI}}}}{6}$$

^c^Mild (99.9–80%), moderate (60–79.9%) and severe (< 60%) according to the recommendation of Blackburn et al.

**Table 2:** Comparison of BIA measurements in cirrhotic patients overall, with and without malnutrition

Variables	Overall	Malnutrition by BCM^a^		p value
	N = 161	Yes N = 95	No N = 66	
		Mean (SD)	Mean (SD)	
Total body water (kg)	34.9 (7.5)	34.2 (8.0)	36.4 (6.1)	0.06
Fat free mass (kg)	45.5 (12.0)	43.43 (13.0)	49.9 (8.4)	< 0.001
Fat free mass index (kg/m^2^)	17 (4.22)	16.5 (4.8)	17.9 (2.5)	0.02
Total body fat percentage	22.2 (10.7)	27 (9.1)	12.7 (6.9)	< 0.001
Body cell mass (kg)^a^	22 (5.8)	20.9 (6.3)	24.0 (4.0)	< 0.001

^a^BCM = F.F.M * 0.29 * LN (5.28), low body cell mass (malnourished) n (%) ≤ 40% of body weight

### Comparison of nutritional assessment by PCM and BIA

There was moderate correlation of PCM score with Body call mass (Pearson correlation coefficient = 0.3 (p value 0.001)). Specificity of BCM for detecting Malnutrition in patients with cirrhosis (presumed gold standard PCM score) is 28% and sensitivity is 60% with a positive predictive value of 60% and a negative predictive value of 39% (Table 3).

**Table 3:** Correlation of PCM score with BIA parameters in patients with cirrhosis

	BMI (kg/m^2^)	FFMI	Total body water (kg)	Fat free mass (kg)	Body fat (%)	Body cell mass^a^ (kg)
Cirrhosis						
PCM Pearson score correlation	0.340	0.24	0.26	0.23	0.25	0.3
p value	0.00	0.005	0.00	0.00	0.00	0.001

Pearson correlation of ≥ 0.3 is considered as optimal correlation

^a^Specificity of BCM for detecting Malnutrition in patients with cirrhosis (gold standard PCM score) is 28% and sensitivity is 60% with a positive predictive value of 60% and a negative predictive value of 39%

## Discussion

We report in this data from 2 tertiary care centers from Karachi, Pakistan that almost two thirds of cirrhotic patients suffer from malnutrition as assessed through PCM score. However when the same population is assessed by BIA, malnutrition is rather underestimated in this cirrhotic patient population. MAC, TSF and 24 h urine creatinine were the main discriminators in differentiating patients with malnutrition and those without it. PCM score and BIA have moderate correlation for assessing malnutrition in this study.

The prevalence of malnutrition in a study on 300 consecutive patients attending outpatient clinics for liver diseases was 75.3%. Out of them 38.3% had moderate or severe malnutrition. We report prevalence of 67% in patients with liver cirrhosis, with 37% falling under moderate to severe category of malnutrition. The reason for this difference might be that the patient population in the former study from Brazil is those with alcoholic cirrhosis and nonalcoholic fatty liver disease, while our patients were largely those suffering from hepatitis B and C. More recently in another study from Brazil on 230 patients with hepatitis B (n = 80) or C (n = 150), 199 (86.5%) patients were well nourished, and 31 (13.5%) were malnourished. This is a much lower prevalence of malnutrition in contrast to our figure of 67%. In fact a large number of participants in this study from Brazil were overweight. We on the other hand did not see such trends in our patient population. In another study on 315 patients from china prevalence of malnutrition (73%) was higher in the cirrhotic group due to viral hepatitis. In a study from India the prevalence of malnutrition was 68%. Our study shows similar pattern of malnutrition in our patients with liver cirrhosis. The seventy percent prevalence of malnutrition is however higher than the reported figure of 52% from the study by Naqvi et al. which might be underestimated due to use of a partially subjective tool of assessment in the later study [25]. These high figures of malnutrition due to viral hepatitis might be due to genotypes of these viruses specific to Asian or South Asian population that could be more virulent. Other factors contributing to malnutrition in patients with liver cirrhosis include inadequate oral intake, metabolic disturbances, malabsorption, and decreased capacity of the liver to store nutrients and dietary restrictions imposed by the family.

International literature shows conflicting results about BIA in cirrhotic patients where one author concluded that it is a reliable bedside tool for the determination of body cell mass in cirrhotic patients with and without ascites [7]. While another author concluded it as a less reliable tool for nutrition assessment in cirrhotic patients with ascites and suggested to use anthropometric measures [8]. We found in this study that BIA under reported malnutrition (61%) compared to PCM score (73%).The reason for this could be the difference in water distribution in cirrhotic due to edema and ascites. Pirlich et al. reports in his study (n = 41) that BIA is a reliable bedside tool for the determination of body cell mass in cirrhotic patients with and without ascites [7]. Our findings are in contrast with this study. The reasons for this might be that the patients in the former study seemed to belong to child class A mainly (Child–Pugh score of 8.1) while at least 40% of our patients belonged to child class B or C, Secondly the patients in that study had cirrhosis due to non-viral etiology while we had patients mainly with viral etiology, which are more sick compared to those with alcoholic liver disease. Thirdly a sample size of 41 indicate an under power study. Similar to our study, a study from Brazil also concluded that Single-frequency electrical bio impedance for body composition analysis in cirrhotic patients must be cautiously used [26].

Although our study suggests that PCM is more sensitive in detecting malnutrition, it however has its limitations like collection of cumbersome biological specimens which patients might not consent for and also this strategy might not be cost effective. The nutritional state assessment in these patients is complicated, and besides anthropometry is based on several other tools in order to be more accurate [12]. We suggest that BIA in combination with mid arm circumferences as a complimentary tool for assessment of malnutrition might be an area for future research. We saw MAC as a discriminator in detecting malnutrition and can be used as a bedside tool for this purpose.

The strength of this study is that two different methods were used for assessment of malnutrition both measures were objective. The use of these objective measures decreases the chances of misclassification bias. A response rate of 80% in the study is considered as optimal.

## Conclusion

We conclude that majority of the patients with liver cirrhosis had malnutrition as determined by PCM score. BIA underscored the malnutrition in this patient population. The correlation of PCM score and BIA was moderate.

## Limitations

There are several limitations in this study, (1) it has limited external validity because it included only patients visiting outpatient clinics in 2 hospitals, hence the results cannot be generalized to the entire population. (2) This is certainly a limitation and future studies ideally should be from population based samples. Being in outpatients setting only patients with well compensated cirrhosis could be recruited while those with advanced disease were not recruited leading to selection bias. (3) Presence of ascites and edema is a limitation for measuring BCM from BIA and also for measuring BMI for PCM score. (4) We did not include information on food intake, type of treatment for cirrhosis which could have been a major determinant in correlating it with malnutrition. (5) While objective measures were used for assessment of malnutrition some degree of observation bias might be involved while measuring MAC and TSF.

## Abbreviations

PCM: protein calorie malnutrition
BIA: bio-electrical impedance analysis
TCF: triceps skin fold thickness
MAMC: mid arm muscle circumference
MAC: mid arm circumference
